# Autistic adults perceive and experience laughter differently to non-autistic adults

**DOI:** 10.1038/s41598-024-56903-8

**Published:** 2024-05-21

**Authors:** Ceci Q. Cai, Sarah J. White, Sinead H. Y. Chen, Marie A. E. Mueller, Sophie K. Scott

**Affiliations:** 1 Institute of Cognitive Neuroscience, University College London, London, WC1N 3AZ UK; 2 Epidemiology and Applied Clinical Research, Division of Psychiatry, University College London, London, W1T 7BN UK

**Keywords:** Human behaviour, Autism spectrum disorders

## Abstract

Human interaction is immersed in laughter; though genuine and posed laughter are acoustically distinct, they are both crucial socio-emotional signals. In this novel study, autistic and non-autistic adults explicitly rated the affective properties of genuine and posed laughter. Additionally, we explored whether their self-reported everyday experiences with laughter differ. Both groups could differentiate between these two types of laughter. However, autistic adults rated posed laughter as more authentic and emotionally arousing than non-autistic adults, perceiving it to be similar to genuine laughter. Autistic adults reported laughing less, deriving less enjoyment from laughter, and experiencing difficulty in understanding the social meaning of other people’s laughter compared to non-autistic people. Despite these differences, autistic adults reported using laughter socially as often as non-autistic adults, leveraging it to mediate social contexts. Our findings suggest that autistic adults show subtle differences in their perception of laughter, which may be associated with their struggles in comprehending the social meaning of laughter, as well as their diminished frequency and enjoyment of laughter in everyday scenarios. By combining experimental evidence with first-person experiences, this study suggests that autistic adults likely employ different strategies to understand laughter in everyday contexts, potentially leaving them socially vulnerable in communication.

## Introduction

Laughter is a universal non-verbal expression of emotion in human interactions^1,2^. Human laughter is highly behaviourally contagious: people are 30 times more likely to laugh when with others than when alone^3^, and laughter can be easily elicited by hearing another’s laughter^4^. The contagious-laughter effect is strongly mediated by social contexts, such as the audience size and the intimacy/familiarity of the relationship^3–5^. Furthermore, contagious laughter is very likely to be unique to humans, as humans can provoke laughter and respond to others’ laughter in the absence of direct physical contact^6^.

Although laughter has long been viewed as a genuine and uncontrolled emotional vocalisation in response to amusement and humour, it predominately occurs in conversation and serves as a communicative tool: people frequently laugh after verbal utterances as signalling friendliness, affiliation, and agreement with others, mediating the meaning of utterances, and regulating the flow of conversation^4,7–10^. The production of laughter varies in the degree of volitional control, emotional content, and authenticity^11–14^; it can be either driven by external stimuli, which are strongly linked to emotional arousal (genuine, spontaneous, involuntary laughter): we laugh uncontrollably to express joy and amusement; or it can be a self-controlled act without any external stimulation, serving as a social signal during interactions (posed, controlled, voluntary laughter): we laugh voluntarily to pass on social meaning and intention in conversations^9,15^. It is worth noting that there is no well-articulated cut-off point between genuine and posed laughter, especially in terms of the degree to which it is associated with a positive internal state. Here, we refer to genuine laughter as spontaneous and involuntarily produced, and to posed laughter as non-spontaneous and voluntarily produced^13,16^. In other words, these two types of laughter are distinguished by their volitional control.

Genuine and posed laughter are acoustically distinct^11,13,16^, and neuroimaging evidence suggests they engage different brain systems during production and perception^11,13,15,17^. In terms of production, posed laughter is likely involved in the volitional speech motor network, controlled by lateral motor cortex regions like the frontal operculum^15,17^. On the other hand, genuine laughter might be governed by the older involuntary vocalisation network that operates through subcortical and brainstem structures. These include the amygdala, thalamic/hypo- and subthalamic areas, and the dorsal/tegmental brainstem^6,12,15^. While the perception of positive nonverbal vocalizations, including laughter, engages the oro-facial mirror networks —indicating a mechanism for mirroring others' emotional expressions—different neural responses have been observed to genuine and posed laughter^12,13,18^. Greater activation has been found in the anterior medial prefrontal cortex (amPFC) and anterior cingulate cortex (ACC) when non-autistic adults passively listen to posed versus genuine laughter. The involvement of mPFC suggests that laughter perception, especially the processing of posed laughter, automatically engages people’s high-level cognitive skills, likely due to mentalizing, in order to understand and interpret social ambiguity, such as the intention and meaning behind the laughter^13,18^.

Additionally, the difference in acoustic features between genuine and posed laughter has an impact on how people perceived its affective properties^11,13,16^. Behavioural evidence indicates that non-autistic adults can differentiate the authenticity between genuine and posed laughter by categorising the former as ‘real’ than compared to the latter^11,13^. They also perceive genuine laughter as significantly more emotionally and behaviourally contagious, as well as more exciting, intense, positive^13^. Furthermore, the authenticity of laughter also leads to processing differences in the communication of emotional and social meaning. Individuals with higher emotional contagion and empathic traits are generally better at judging the authenticity of laughter^19^. This further supports the socio-emotional determinants of laughter processing^12^. Collectively, laughter varies depending on its authenticity, and accurately understanding the social meaning and intention of laughter is crucial when people use laughter socially, from establishing and maintaining social bonds and relationships, to regulating emotions^12,20^. From an evolutionary perspective, laughter has been proposed to promote group cohesion and social bonding, as well as building rapport in human interaction^20–23^. Since laughter is an important and a complex socio-emotional signal, it is critical to investigate laughter behaviour and experience in autistic people, as they experience difficulties in social communication^24^.

To date, research into laughter production and perception in the autistic people is rare. Limited evidence suggests a different profile of laughter behaviour likely exits in autistic people, especially in autistic children compared to their peers^25–29^. For instance, compared to children with Down Syndrome, autistic children have been observed to produce significantly more unshared laughter during play and show less response (neither looking up nor smiling) to their parents’ laughter. Furthermore, fewer autistic children were reported to join in others’ laughter or elicit laughter from others by clowning or teasing, even though there was no group difference in laughter frequency based on parental reports^25^. Additionally, autistic children were found to primarily produce ‘voiced laughter’ in social play, which is often associated with the producer’s positive affective state^26,30^. In contrast, they exhibited relatively limited ‘unvoiced laughter’, which typical-developing children seem to rely on heavily during social interactions. The usage of ‘unvoiced laughter’ tends to increase with age and is influenced by social contexts^5,26^. Regarding laughter processing, typical-developing children rated ‘Tom and Jerry’ cartoons as more enjoyable and laughed more when a laugh track accompanied the cartoon than when it was absent. In contrast, autistic children rated cartoons with a laughter track as less enjoyable and produced fewer laughs and smiles^27^. Interestingly, we reported that adding genuine and posed laughter to spoken ‘dad jokes’ resulted in the jokes being rated as ‘funnier’. The same pattern of implicit laughter processing was seen in autistic and non-autistic adults: the addition of laughter increased the perceived funniness of the jokes, moreover, jokes paired with genuine laughter were perceived as funnier than with the addition of posed laughter^31^. However, it is still unclear whether autistic adults process laughter differently from non-autistic adults when rating the two different kinds of laughter more explicitly. In other words, it is unclear whether autistic adults can differentiate between genuine and posed laughter in the same way that non-autistic adults can.

In addition to experimental data, self-report data is valuable in illustrating the experience of laughter. Autism research has recently shifted focus to include the first-person experience of autistic people more often, emphasizing the significance of understanding autistic adulthood and supporting their overall well-being^32,33^. However, no research has yet employed self-report questionnaires to explore the first-hand experience of laughter in autistic adults. Therefore, it is crucial to comprehend the role of laughter in the daily lives of autistic adults to gain a deeper understanding of the distinct profile of nonverbal social communication in this population. If a discrepancy arises between existing empirical data and self-report data, it could indicate that autistic people have limited insight into their own social communication, behaviour and cognition.

Despite laughter's vital role in establishing and nurturing social relationships, few questionnaire studies have investigated individual laughter experiences. Some of these questionnaires focused more on humour (e.g., SHRQ; Ref.^34^; CHS; Ref.^34,35^), while others focused on measuring abnormal and unusual laughter preferences (e.g., PhoPhiKat-45; Ref.^36^), including personal fear and joy of being laughed at (gelotophobia and gelotophilia, respectively), as well as personal joy derived from laughter at others (katagelaticism). Therefore, we used a novel laughter questionnaire which focused on people’s self-reported laughter experiences in everyday life^37^. The 30-item laughter perception and production questionnaire (LPPQ) assesses people’s daily laughter behaviours in both production level (Frequency of laughter and Usage of laughter) and perception level (Understanding of laughter and Liking of laughter)^37^.

In the current study, we aim to provide the first detailed picture of how autistic people explicitly process genuine and posed laughter, as well as their laughter experience in everyday life. Autistic and non-autistic (NA) adults, matched for age, gender and IQ, participated in an in-lab experiment where they rated genuine and posed laughter stimuli across four ratings blocks: authenticity, contagion, valence and arousal. We intentionally separated the ratings into four blocks to independently assess the processing of different types of laughter for each rating. Our aim was to determine whether a different processing pattern exits between autistic and NA adults within each rating block. Notably, the authenticity block was always presented first to determine if participants could differentiate between genuine and posed laughter based on their initial presentation. The order of the other three blocks was randomized, as we aimed to assess whether their judgment of perceptual affective properties was influenced by different types of laughter. In a subsequent phase, the LPPQ was administrated to autistic and non-autistic adults, matched for age, gender, and IQ, through an in-lab experiment. A supplementary online experiment was conducted to replicate the LPPQ findings. Based on the evidence of different patterns of laughter production and perception between autistic people and their peers, we hypothesised that autistic adults would show a different pattern of perceptual judgement relative to non-autistic adults, specifically, that they would perform less well in differentiating the authenticity between genuine and posed laughter relative to non-autistic adults, and would also show a different pattern in perceiving the affective properties of laughter. Additionally, we hypothesised that self-reported laughter in both production and perception would differ between autistic adults and non-autistic adults. Furthermore, this difference would be consistent in both in-lab groups and online groups.

## Results

Here, we first present the results from the explicit ratings of laughter, followed by the in-lab and online results from the self-reported laughter questionnaire: LPPQ. Given the small overlap in samples between the in-lab experiments, we also conducted an exploratory analysis to determine if participants’ ability to distinguish between genuine and posed laughter could account for their self-reported perception and production of laughter in daily life across the four LPPQ components. Data were analysed in IBM SPSS Statistics (version 27) and RStudio Team (2020). Reported *p* values are two-tailed. Prior to statistical analysis, both behavioural and questionnaire data were plotted to investigate distributions and identify outliers and missing data (see Supplementary Information for details).

### Explicit processing of laughter

Both autistic (*n* = 25) and non-autistic (*n* = 25) participants completed an in-lab behavioural rating of genuine and posed laughter stimuli across four rating blocks: authenticity, contagion, valence, and arousal. Therefore, a 2 × 2 mixed measures analysis of variance (ANOVA) was conducted on each scale of the perceptual affective properties of laughter, including the type of laughter (genuine vs posed) as the within-subject factor and participant group (Autism vs NA) as the between-subjects factor. Greenhouse–Geisser corrections were used.

On the authenticity rating, there was a significant main effect on the type of laughter, *F [1, 48]*  = 254.119, *p* < 0.001, ${\eta }_{p}^{2}$ = 0.841, indicating that participants found genuine laughter (*M* = 5.593, *SD* = 0.696, *SEM* = 0.098) to be more authentic than posed laughter (*M* = 3.607, *SD* = 0.838, *SEM* = 0.119). There was no significant main effect of group,* F [1, 48]* = 3.656, *p* = 0.062, ${\eta }_{p}^{2}$= 0.065, but there was a significant interaction effect between laughter type and group, *F [1, 48]* = 6.261, *p* < 0.05, ${\eta }_{p}^{2}$= 0.115. Post hoc analysis indicated that the non-autistic group (*M* = 3.289, *SD* = 0.791, *SEM* = 0.158) rated posed laughter as significantly less authentic than the autistic group (*M* = 3.925, *SD* = 0.773, *SEM* = 0.155), *t *(48) = 2.874, *p* < 0.01. However, there was no significant difference for ratings of genuine laughter between non-autistic (*M* = 5.587, *SD* = 0.699, *SEM* = 0.140) and autistic (*M* = 5.599, *SD* = 0.706, *SEM* = 0.141) participants, *t*(48) = 0.006, *p* = 0.951.

On contagion rating, there was a significant main effect of type of laughter, *F [1, 48]* = 234.712, *p* < 0.001, ${\eta }_{p}^{2}$= 0.830, indicating that participants found genuine laughter (*M* = 5.129, *SD* = 0.855, *SEM* = 0.121) to be more contagious than posed laughter (*M* = 3.113, *SD* = 0.903, *SEM* = 0.128). There was no significant main effect of group, *F [1, 48]* = 0.190, *p* = 0.665, ${\eta }_{p}^{2}$ = 0.004, and no significant interaction effect between laughter type and group, *F [1, 48]* = 3.341, *p* = 0.074, ${\eta }_{p}^{2}$= 0.065.

On valence rating, there was a significant main effect of type of laughter, *F [1, 47]* = 214.942, *p* < 0.001, ${\eta }_{p}^{2}$= 0.821, indicating that participants felt the sound of genuine laughter (*M* = 5.718, *SD* = 0.524, *SEM* = 0.075) reflected a more positive emotion than the sound of posed laughter (*M* = 3.933, *SD* = 0.693*, SEM* = 0.099). There was no significant main effect of group, *F [1, 47]* = 1.062, *p* = 0.308, ${\eta }_{p}^{2}$ = 0.022, and no significant interaction effect between type and group, *F [1, 47]* = 3.471, *p* = 0.069, ${\eta }_{p}^{2}$= 0.069.

**Figure 1:**
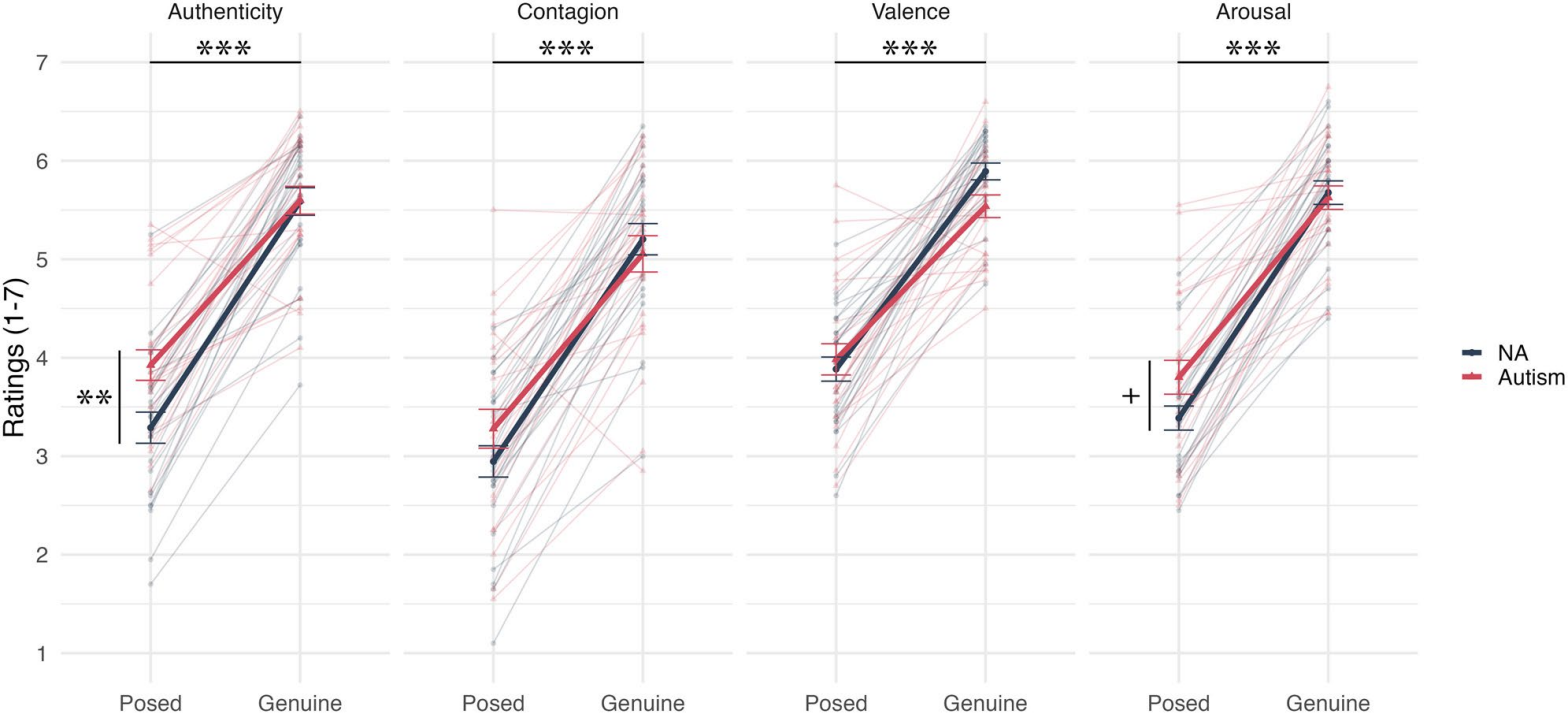
Explicit ratings of posed and genuine laughter across authenticity, contagion, valence and arousal between autistic and non-autistic adults. Each light colour line represents an individual’s average ratings for posed and genuine laughter; Each dark colour line represents average ratings of laughter across a group; *NA* Non-autistic; Error bars: ± 1 SE; Main effects: ***p < .001; Interaction effects: **p < .01, ^+^*p* = .055.

On the arousal rating, there was a significant main effect on the type of laughter, *F [1, 48]* = 360.197, *p* < 0.001, ${\eta }_{p}^{2}$= 0.882, indicating that participants felt the sound of genuine laughter (*M* = 5.651, *SD* = 0.591, *SEM* = 0.084) reflected stronger emotional arousal than the sound of posed laughter (*M* = 3.594, *SD* = 0.768, *SEM* = 0.109). There was no significant main effect of group, *F [1, 48]* = 1.334, *p* = 0.254, ${\eta }_{p}^{2}$= 0.027, but there was a significant interaction between laughter type and group, *F [1, 48]* = 4.616, *p* < 0.05, ${\eta }_{p}^{2}$= 0.088. Post hoc analysis indicated that the non-autistic group (*M* = 3.387, *SD* = 0.612, *SEM* = 0.123) rated posed laughter as less emotionally arousing than the autistic group, with a borderline significant effect (*M* = 3.801, *SD* = 0.860, *SEM* = 0.172), *t*(48) = -1.965, *p* = 0.055. However, there was no significant difference in ratings of genuine laughter between non-autistic (*M* = 5.676, *SD* = 0.596, *SEM* = 0.119) and autistic (*M* = 5.625, *SD* = 0.596, *SEM* = 0.119) participants, *t*(48) = 0.303, *p* = 0.763. See Fig. 1.

Although two of these interactions were significant and two were borderline, we found that the four different types of rating were correlated to each other within genuine laughter and within posed laughter across the whole sample. Spearman’s rank correlations authenticity, contagion, valence and arousal ratings of genuine laughter showed significant positive correlations (all *r*(48) > 0.40, *p* < 0.005). On the ratings of posed laughter, significant positive correlations were again found between the four rating scales (all *r*(48) > 0.48, *p* < 0.001). All *p*_*FDR*_ < 0.05. This indicates that the participants were performing similarly across all the different rating scales, perhaps due to these different rating scales tapping into shared properties of laughter.

### Self-reported laughter experience—LPPQ

**Figure 2:**
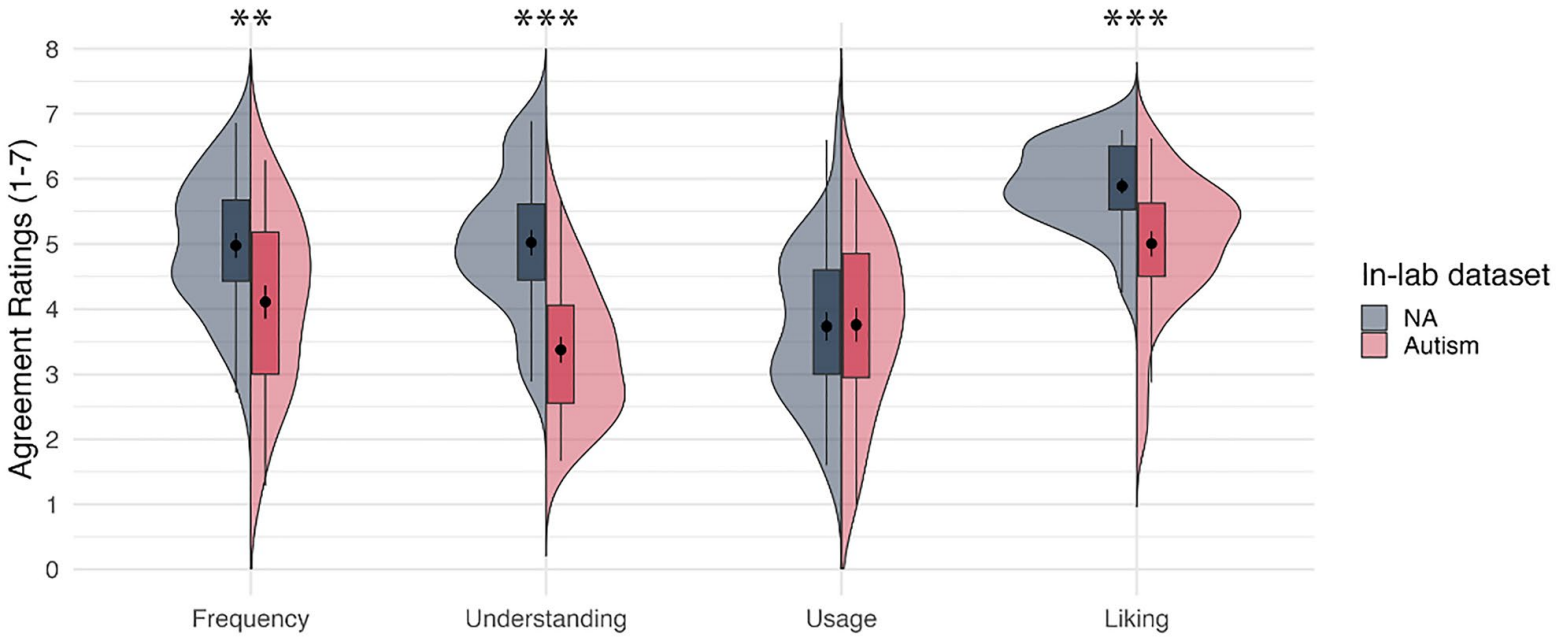
Average ratings for agreement on four components between the Non-autistic group and Autistic group from the in-lab dataset. *NA*  Non-autistic, Dot with short line inside the boxplot = mean ± 1SE; Significance code: *p* < .01 = **, *p* < .001 = ***.

Similar group differences on the four components of LPPQ were found in both the in-lab and online datasets. For the in-lab dataset, independent sample t-tests indicated that there was a significant difference between the autism (*n* = 28) and NA (*n* = 30) group on Frequency, *t*(56) = 2.761, *p* < 0.01, Understanding, *t*(56) = 5.888, *p* < 0.001, and Liking, *t*(56) = 3.989, *p* < 0.001, but not on Usage, *t*(56) = − 0.072, *p* = 0.943. Since we found group difference in levels of depression, additional ANCOVA analyses was conducted, controlling for BDI score. As before, significant group differences were found on Frequency, *F*(1, 55) = 8.427, *p* = 0.005, Understanding, *F*(1, 86) = 32.640, *p* < 0.001, and Liking, *F*(1, 86) = 30.373, *p* < 0.001, but there was no significant difference on Usage, *F*(1, 86) = 0.389, *p* = 0.535. See Fig. 2.

**Figure 3:**
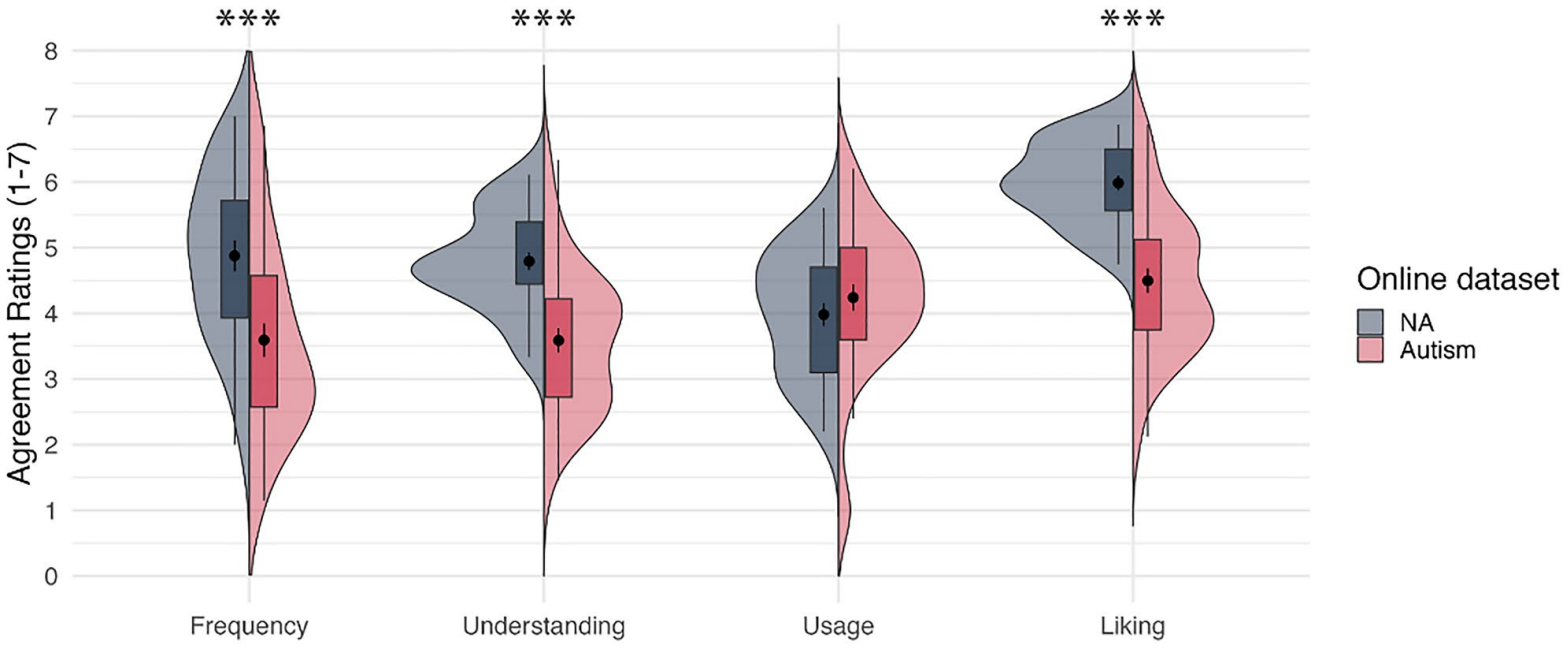
Average ratings for agreement on four components between the Non-autistic group and Autistic group from the online dataset. NA = Non-autistic, Dot with short line inside the boxplot = mean ± 1SE, Significance code: *p* < .001 = ***.

In the subsequent analysis on online datasets, we found similar patterns. Independent samples t-tests indicated that there was a significant difference between the autism (*n* = 35) and NA (*n* = 31) groups for Frequency, *t*(85) = 3.971, *p* < 0.001, Understanding, *t*(58.831) = 5.688, *p* < 0.001, and Liking, *t*(54.942) = 6.328, *p* < 0.001. However, no significant difference between the groups was found for Usage, *t*(85) = 1.258, *p* = 0.212. Since we found group difference in baseline mood, additional ANCOVA analyses were conducted controlling for mood. As before, a significant difference was found for Frequency, *F*(1, 63) = 7.346, *p* = 0.009, Understanding, *F*(1, 63) = 23.984, *p* < 0.001, and Liking, *F*(1, 63) = 35.358, *p* < 0.001, but not for Usage, *F*(1, 63) = 0.814, *p* = 0.370. See Fig. 3.

### Correlation between laughter differentiation and laughter experience

**Figure 4:**
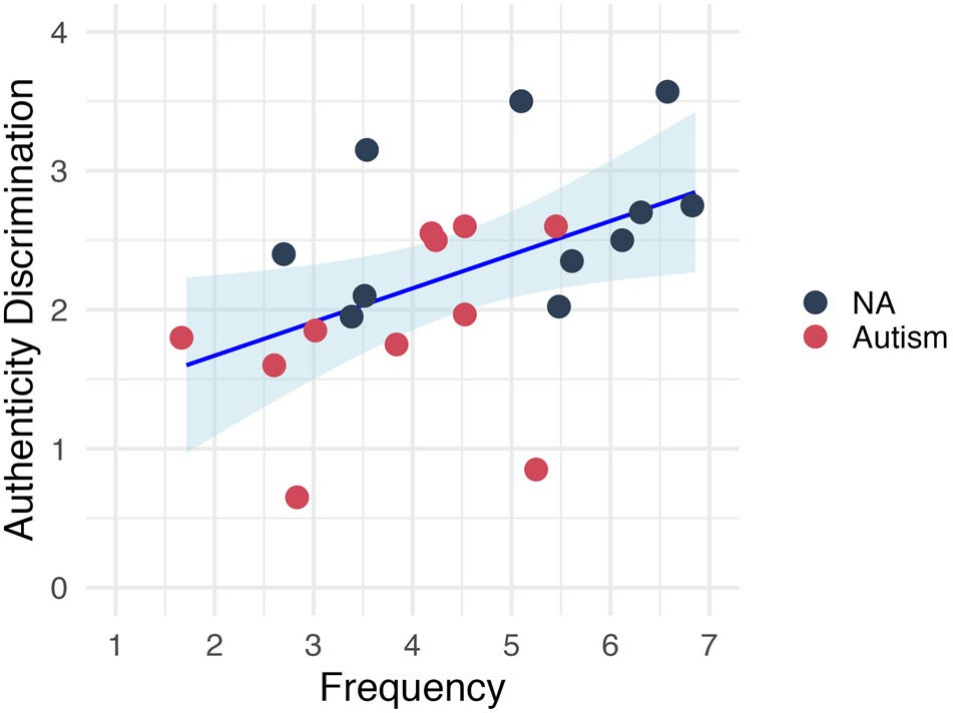
Correlation between self-reported laugh frequency and the ability in discriminating the authenticity of laughter. Authenticity Discrimination = individual’s average ratings of genuine laughter minus posed laughter on authenticity scale. *NA*   Non-autistic.

There was an overlap in participants between the in-lab behavioural ratings and in-lab questionnaire datasets (NT *n* = 11; Autism *n* = 11; see Table S5). We therefore conducted a further exploratory analysis to investigate whether participants’ ability to discriminate genuine and posed laughter (individual’s average ratings of genuine laughter minus posed laughter on each of the 4 rating scales) could correlate with their self-reported perception and production of laughter in everyday life on each of the 4 LPPQ components. Spearman’s rank correlations were conducted across all the participants; a positive correlation was found between the ability to discriminate the authenticity of laughter and the frequency of laughter production in daily life, *r*(20) = 0.59, *p* < 0.001 (*p*_*FDR*_ = 0.058). See Fig. 4. All other correlations between the other rating scales and the other questionnaire components were not significant.

## Discussion

The aim of the present study was to explore the perceptual differences between genuine and posed laughter in autistic and non-autistic adults. Concurrently, we sought to investigate differences in self-reported laughter experiences. The explicit ratings of the affective properties of laughter revealed that autistic adults can distinguish between genuine and posed laughter, albeit to a lesser extent compared to non-autistic adults. However, autistic adults tended to perceive posed laughter as more authentic and more emotionally arousing than non-autistic adults. Regarding self-reported laughter experiences, both the in-lab dataset and supplementary online dataset consistently showed differences between groups. Non-autistic adults reported laughing more frequently (Frequency), having a greater enjoyment for laughter (Liking), and understanding others' laughter better (understanding). However, both autistic and non-autistic adults reported using laughter for its positive social effects (Usage) to the same extent. Moreover, those individuals who were weaker at differentiating genuine and posed laughter also reported using laughter less frequently in daily life. Overall, we found differences in laughter perception and personal experience between autistic and non-autistic adults, which maybe functionally connected.

Both autistic and non-autistic adults rated genuine laughter as significantly more authentic, contagious, positive and emotionally arousing than posed laughter. These findings support previous evidence that non-autistic adults perceive genuine laughter as more authentic than posed laughter, and that the authenticity of laughter affects its perceptual affective properties^11,13^; these same perceptual patterns have been found across the lifespan^14^. In the current study, we extend this knowledge to the autistic population, revealing that autistic adults, like their non-autistic peers, experience perceptual and affective differences between genuine and posed laughter. This is notable as a relative social strength in autism.

Despite this social ability, we found a subtle decrease in the degree to which autistic adults differentiated between these different types of laughter. Interestingly, autistic adults rated posed laughter as significantly more authentic and emotionally arousing than non-autistic adults, meaning posed laughter was perceived as being more like genuine laughter. These findings support our hypothesis that autistic adults exhibit some degree of perceptual difference in determining the authenticity of laughter. This could be due to autistic people having a different laughter production pattern: autistic children primarily produced ‘voiced’ laughter which is linked to their positive affect and high emotionally arousal^5,26,30^. This preference for expressing laughter primarily in response to positive internal states may impact the perceptual patterns of autistic people, causing them to perceive genuine and posed laughter with a more similar level of emotional arousal that reflects their internal experience of positive affect.

Our findings are in line with previous literature. Autistic adults with high IQs demonstrate difficulty with differentiating the authenticity of positive facial expressions, such as smiles^38^. Importantly, the ability to distinguish a genuine from a posed smile is associated with the ability to understand and attribute another’s mental state, as a posed smile can indicate the pretence of happiness or pleasure^38^. Similarly, neuroimaging studies consistently find the involvement of the medial prefrontal cortex (mPFC), which is involved in the mentalizing network^39^, during the perception of laughter in non-autistic people^13,18,40^. Further, mPFC activity has been found specifically in response to posed vs genuine laughter^13^. It is therefore possible that the subtle perceptual difference we found is a result of autistic people’s difficulties in attributing mental states to others when hearing posed laughter. However, it should be noted that autistic adults were still able to explicitly differentiate between genuine and posed laughter. This may be possible due to the inherent acoustic differences between genuine and posed laughter^11,41^, without a full understanding of the meaning of these differences. This is further supported by our supplementary finding that only the spectral centre of gravity significantly predicts the authenticity ratings in both autistic and non-autistic adults across all acoustic features.

This is the first study to explore autistic adults' personal experience of laughter in everyday life. Our findings of reduced frequency, enjoyment and social understanding of laughter align with the existing observations of autistic children^25–27,29^. Previous studies have found autistic children predominantly produce “voiced” laughter to express their genuine positive emotions^26,30^, and rarely laugh in response to social events or use laughter as a social signal during social play relative to their peers^25–27^. Since laughter primarily serves as a communicative tool in conversations, it is likely that autistic adults produce a typical amount of genuine laughter, but tend to laugh less when using it as a social cue or in response to social situations, leading to their report of a lower frequency of laughter production compared to non-autistic adults. This interpretation is supported by our finding that those individuals who reported laughing least in everyday life were the same individuals who differentiated least between genuine and posed laughter. Previous studies also show that autistic children rated Tom and Jerry cartoons as less enjoyable when a laugh track was added, and they laughed less when watching the cartoon with a laughter track compared to their typically developing peers^27^. This is in line with our autistic adults’ report of less enjoyment of laughter, and may also be explained by our finding of lower understanding of laughter. Since autistic adults experience difficulty in interpreting the meaning and intention behind other’s laughter, particularly in socially ambiguous situations, this may make other people’s laughter less enjoyable.

Surprisingly, we found no difference in the use of laughter for its positive social effects between non-autistic and autistic adults. Since our participants were high IQ autistic adults, they may not be representative of the entire autistic population and certainly differ from the autistic children tested in previous studies, who used laughter less as a social signal. Indeed, it has been suggested that many autistic adults may engage in social camouflaging, adapting and conforming to social environments predominantly shaped by non-autistic people^42,43^ This social camouflaging can be beneficial in establishing relationships with non-autistic people^42^. Therefore, autistic people may engage in, but perhaps experience delays in, developing strategies and behaviours related to laughter norms established by non-autistic people, such as intentionally using laughter for social purposes in daily interactions. Although we found some differences in self-reported laughter experiences between groups, it is important to note that people may not be fully aware of their use of posed laughter. This lack of awareness could introduce biases in the self-reported data of the current findings. Additionally, the self-reported questionnaire might have been influenced not only by individual differences in self-awareness but also by reputation management. Autistic adults may have limited insights into their own laughter behaviour in everyday life. For instance, they might believe they are proficient in using and understanding laughter in social situations, while their actual behaviour suggests otherwise. Conversely, some autistic adults might be aware of their challenges and aim to overcome them, which could influence their self-reports based on these aspirations.

Further research is necessary to address the limitations of the present study. Given that we use auditory stimuli and participants are required to rate based on the subtle differences between genuine and posed laughter within 3-s windows for each stimulus, the task performance is highly sensitive to a quiet testing environment. Consequently, we refrained from replicating the explicit ratings in online tests. First, a future study should use a single, large sample, assessing the same set of participants for both explicit ratings of laughter and self-reported laughter experience. This design, coupled with subsequent analysis, would provide more robust conclusions based on our current findings. It would also offer a more comprehensive profile of the relationship between perception and experience of laughter, and to undercover which aspects of the everyday life difficulties are most closely associated with the inability to differentiate laughter. Second, our rating scales may have lacked sensitivity, making them less ideal for measuring laughter-contagion effects and valence, the two aspects of laughter that didn’t significantly differ between the groups. Future studies could therefore employ alternative methods, such as electromyography, to measure contagion effects more accurately. Additionally, assessing participants' meta-cognitive abilities regarding their ratings (i.e., asking participants to rate their confidence in distinguishing between genuine and posed laughter) would provide more valuable insights in addition to the current experiment. Third, autistic people are known to have higher rates of co-occurring mental health or psychiatric conditions^44^, such as anxiety and depression, which may affect their everyday laughter experience. In the current study, we only measured participants’ depression level and/or current mood. However, it is important to take into account the potential influence of mental health factors and control for these in future studies to understand the contribution of these conditions and ensure a stronger interpretation of the differences in everyday laughter experience between autistic and non-autistic adults.

Overall, we found subtle differences between autistic and non-autistic adults in laughter perception and personal experience. Autistic adults can discriminate genuine and posed laughter but judge posed laughter to be more like genuine laughter than non-autistic adults. This lines up with the personal experience of autistic adults, who report struggling to understand the social meaning of others’ laughter. In addition, this perceptual pattern may result in using and enjoying laughter less in everyday life and may indicate that autistic adults use different strategies to understand laughter.

## Method

### Explicit rating of laughter

#### Participants

In total, 26 autistic adults (five females) and 27 non-autistic (NA) adults (7 females) took part in this study. All participants in the autistic group had a diagnosis of autism spectrum disorder (*n* = 5) or Asperger syndrome (*n* = 21) from a qualified clinician, with 12% reporting an additional diagnosis of another developmental disorder: dyslexia (*n* = 1), ADHD (*n* = 1) and dyspraxia (*n* = 1). The Autism Diagnostic Observation Schedule (ADOS; Ref.^45^) was administered to verify diagnoses. In total, 10 participants met the criteria for autism and 11 more for autism spectrum on the ADOS. The remaining five scored below the threshold. However, they were retained in the sample because all reported significant social difficulties in everyday life. Specifically, ﻿three of them had an AQ score above the recommended cut-off of 32^46^, one scored 24, and another did not complete the AQ. Additionally, all displayed symptoms consistent with autism on the ADOS, albeit subthreshold.

However, three participants (1 from the Autistic group, 2 from the NA group) were excluded from further analysis as they were considered outliers on the explicit rating task (see Supplementary Information for details). The resulting groups were comparable on gender (*χ*^2^(1) = 0.439, *p* = 0.508), age (*t*(48) = 0.525, *p* = 0.602) and verbal (*t*(48) = 1.196, *p* = 0.237) and performance (*t*(48) = 1.487, *p* = 0.143) IQ, as measured by either the Wechsler Adult Intelligence Scale (WAIS-III UK; Ref.^47^) or Wechsler Abbreviated Scale of Intelligence (WASI-II; Ref.^48^), and also differed on the Autism-Spectrum Quotient (AQ; Ref.^46^) (*t*(48) = 9.819, *p* < 0.001). See Table 1.

**Table 1:** Demographic details of the participants in explicit rating task.

	Autism	NA
*N* (male:female)	25 (20:5)	25 (18:7)
Age (years)	34.680 (7.793)	33.280 (10.826)
Verbal IQ	115.480 (10.162)	118.960 (10.406)
Performance IQ	110.200 (14.620)	116.000 (12.900)
AQ^a^	33.792(8.325)	13.880 (5.674)
ADOS total	8.720 (3.434)	N/A
Communication	3.320 (2.393)	N/A
Social	5.800 (2.415)	N/A

Values are given as mean (standard deviation).

*NA* non-autistic, *AQ* autism-spectrum quotient.

^a^One autistic participant did not complete the AQ questionnaire.

#### Laughter stimuli

The laughter stimuli (40 in total) consisted of 20 genuine and 20 posed laughs. The duration of each stimulus was edited and cut into complete laughter sound clips from 2 to 2.99 s (average duration = 2.51 s; *SD* = 0.36; range = 1.7 to 3.14 s). The laughter stimuli were selected from the emotional vocalisation dataset (100 in total) used in a previous study. See^14,31^.

Briefly stated, the genuine and posed laughter were recorded using the method previously validated in behavioural and neuroimaging experiments^11,13,18,31,49^. The laughter was generated by six adults who were not professional actors (aged between 23 to 46 years; three females) and recorded using professional equipment in a sound-proof, anechoic chamber at University College London. To elicit genuine laughter, each speaker viewed videos on a computer screen whilst wearing headphones, which had been identified beforehand as amusing to that participant. The emotional experience was described positively by speakers during and after the recording session of genuine laughter. To produce posed laughter, speakers were asked to generate laughter “on demand” under their volitional control without any external stimulation. They were also encouraged to make it sound natural and positive. Importantly, speakers were always asked to produce posed before genuine laughter to avoid the positive emotional states associated with genuine laughter from affecting the production of posed laughter. The raw audio files were down sampled at 44100 Hz to mono.wav files with 32-bit resolution. Individual files were prepared for each vocalisation from each speaker by visually identifying the onset and offset of each event in their oscillograms. All files were then normalised for root-mean-square (RMS) amplitude using PRAAT^50^. From the original 100 laughter stimuli, a pilot perceptual validation experiment was conducted to select the best examples from the genuine and posed laughter stimuli set. Thirty native British speakers rated each stimulus on four parameters (authenticity, emotion, frequency, and control) using a 7-point rating scale. Based on the authenticity ratings, 20 genuine laughter stimuli with the highest authenticity scores and 20 posed laughter stimuli with the lowest scores were chosen for the current experiment. For a comprehensive overview, refer to^14^.

A range of acoustic parameters was extracted on the 40 laughter stimuli by using PRAAT^50^. Independent t-tests indicated that genuine and posed laughter were significantly different in pitch (genuine, *M* = 404.654 Hz, *SD* = 51.446, posed, *M* = 272.534 Hz, *SD* = 74.889, *t*(34) = 6.503, *p* < 0.001), spectrum centre of gravity (Hz) (genuine, *M* = 1293.041 Hz, *SD* = 450.665, posed, *M* = 873.716 Hz, *SD* = 272.458, *t*(38) = 3.561, *p* = 0.001), jitter (local) (genuine, *M* = 2.746 Hz, *SD* = 0.943, posed, *M* = 4.118 Hz, *SD* = 0.915, *t*(38) = − 4.672, *p* < 0.001) and Mean harmonics-to-noise ratio (HNR) (genuine, *M* = 8.110, *SD* = 2.627, posed, *M* = 6.121, *SD* = 2.007, *t*(38) = 2.690, *p* < 0.001). They were matched on duration (genuine, *M* = 2530 ms, *SD* = 0.385; posed, *M* = 2382 ms, *SD* = 0.362) and other measures — root-mean-square (RMS), intensity (dB), standard deviation of pitch (Hz), spectral standard deviation (Hz), fraction of locally unvoiced frames, and shimmer (local, dB).

#### Experimental design

**Figure 5:**
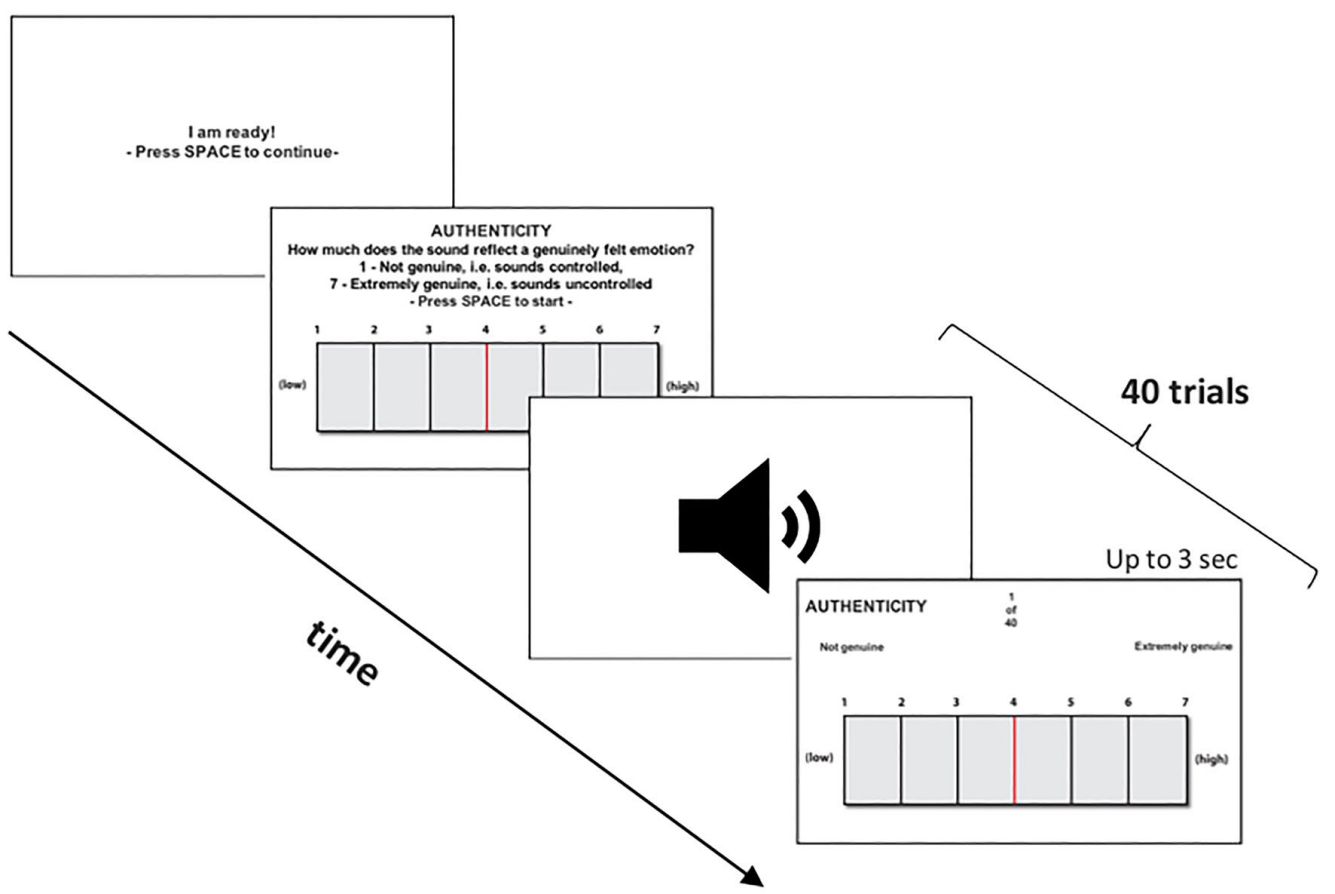
Experimental design for explicit ratings of laughter. The sample is illustrated by the authenticity block. In total, participants rated 40 laughter stimuli across four rating blocks.

All participants rated 20 genuine and 20 posed laughter stimuli across four distinct 7-point rating blocks: Authenticity, Contagion, Valence, and Arousal, resulting in a total of 160 trials. Participants therefore heard each stimulus four times, once in each block. Within each block, the order of laughter stimuli was randomised (See Fig. 5).

- Authenticity: ‘How much does the sound reflect a genuinely felt emotion?’ 1—Not genuine, i.e. sounds controlled, 7—Extremely genuine, i.e. sounds uncontrolled.
- Contagion: ‘How much does hearing the sound make you feel like joining in and/or feeling the emotion?’ 1—Not at all, i.e. it does not make me feel like joining in and/or feeling the emotion, 7—Extremely, i.e. it makes me feel like joining in and/or feeling the emotion.
- Valence: ‘How much does the sound reflect a positive or negative emotion?’ 1—Highly Negative, i.e. the person has the experience of extreme discomfort, 7- Highly Positive, i.e. the person has the experience of extreme pleasure.
- Arousal: ‘How much does the sound reflect emotional arousal?’ 1—Calm, i.e. the person who made this sound is feeling sleepy and with no energy, 7—Aroused, i.e. the person who made this sound is feeling alert and energetic.

The Authenticity block was consistently presented first, with the subsequent three blocks counterbalanced among participants. This sequence was designed to prompt participants to rate laughter authenticity based on their initial instinct, rather than a prolonged evaluation, since they were not informed about the two types of laughter. Participants were required to press the spacebar to proceed to the next rating block, allowing for a break between blocks (See Fig. 5). The entire task was delivered on a laptop using MATLAB software (version R2014a^51^), with Psychtoolbox^52^ and took a maximum of 16 min.

#### Testing procedure

One experimenter administered the task in a testing room. At the beginning of the session, all participants were given a brief explanation of the study. The experimenter remained in the testing room throughout the test session. All participants were instructed to rate laughter stimuli across four scales; however, they were not told that there were two types of laughter. Additionally, participants were asked to sit directly in front of the laptop and wear headphones (Sennheiser headphones HD 221). Participants were instructed that, once the laughter stimuli were played through the headphones, they should respond to each rating as soon as possible. After each laughter stimulus offset, they had a three-second window to response using the keyboard. Before testing began, participants confirmed their understanding of the experiment and had the opportunity to ask questions. The experiment started with the display of task instruction, followed by a practice session in which two laughter stimuli (one genuine and one posed laughter stimulus) were rated across all four rating blocks, leading into the main task. After the experiment, participants were asked if they had additional questions and were compensated for their participation. Participants were also encouraged to contact the researchers if they had any further questions.

### Self-reported laughter experience

#### In-lab participants

In total, 28 autistic adults and 67 non-autistic (NA) adults, who were native English speakers, were recruited from local participant databases. All autistic participants had received a diagnosis of Autism Spectrum Disorder (*n* = 12) or Asperger syndrome (*n* = 16) from a qualified clinician. The Autism Diagnostic Observation Schedule (ADOS-2, module 4; Ref.^45^) was used to verify the diagnosis of 26 autistic participants. Of these, eighteen of them either met the ADOS criteria for autism (*n* = 13) or autism spectrum (*n* = 5). The remaining eight scored below the threshold but were retained within the group: five of them reported an AQ score above the 32-cut-off point and one scored 31^46^; additionally, they all reported significant social difficulties in everyday life.

NA participants were over-recruited on purpose to provide a match group as close as possible to the autistic group. The full NA group was younger, and females were relatively overrepresented. We therefore systematically excluded all females aged below 31 (*n* = 26) and all males aged below 25 (*n* = 11). The groups were comparable on sex (*χ*^2^(1) = 0.646, *p* = 0.421), age (*t*(56) = 0.722, *p* = 0.473), verbal (*t*(56) = 0.803, *p* = 0.426) and full-scale (*t*(56) = 0.631, *p* = 0.531) IQ, as measured by the Wechsler Adult Intelligence Scale (WAIS-III/IV; Ref.^47,53^). As expected, the groups differed in their self-report of autistic traits (*t*(56) = 6.876, *p* < 0.001), measured by the Autism-Spectrum Quotient (AQ; Ref.^46^), and differed in their self-reported depression level (*t*(56) = 2.996, *p* = 0.004 < 0.01), measured by the Beck Depression Inventory (BDI; Ref.^54^). Before filling in the laughter questionnaire, participants were asked to complete the 10-item Positive Affect Schedule (PAS-10; Ref.^55^), which served as a baseline measure of emotion and mood (scores from 1–5, higher scores represent higher level of positive affect at the moment). They were instructed to ‘indicate to what extent you feel this way right now, that is, at the present moment’. There was no significant difference in the baseline mood between the NA group and the autistic group (*t*(56) = -1.202, *p* = 0.235). Full details of the two groups are given in Table 2.

**Table 2:** Demographic details of the participants in in-lab questionnnaire task.

	Autism	NA
*N* (male: female)	28 (23:5)	30 (22:8)
Age (years)	34.143 (6.364)	33.000 (5.693)
Verbal IQ	115.214 (15.847)	118.233 (12.724)
Full Scale IQ	113.929 (16.615)	116.533 (14.827)
AQ	32.357 (10.228)	16.067 (7.719)
BDI	13.500 (7.876)	7.667 (6.945)
PAS-10	2.982 (0.727)	3.200 (0.653)
ADOS total ^a^	8.962 (4.142)	N/A
Communication	2.692 (1.715)	N/A
Social	6.269 (3.027)	N/A

Values are given as mean (standard deviation).

*NA* Non-autistic, *AQ* autism-spectrum quotient, *BDI* The beck depression inventory, *PAS-10* 10-item Positive Affect Schedule.

^a^Two autistic participants did not complete the ADOS.

#### Online participants

Under COVID-19 testing restrictions, we further recruited 52 NA adults (37 females; average age = 24.072, *SD* = 4.423) and 37 autistic adults via Prolific (www.prolific.co) to replicate the in-lab findings. NA adults were over-recruited to provide a match group as close as possible to the autistic group. As they were generally younger and of lower verbal ability, all participants aged below 28 and with a verbal task score below 60 were excluded (*n* = 21). Two autistic participants were excluded from the autistic group because they self-identified as autistic without receiving any clinical diagnosis and their AQ-10^56^ score was below the cut-off point of 6.

The groups were comparable on sex (*χ*^2^(1) = 0.649, *p* = 0.421), age (*t*(62.312) = 1.017, *p* = 0.313), verbal (*t*(64) = 0.809, *p* = 0.422) and non-verbal (*t*(64) = 0.047, *p* = 0.963) abilities, as measured by the Spot-the-Word test (StWt; Ref.^57^ and the Matrix Reasoning Item Bank (MaRs-IB; Ref.^58^) respectively. As expected, the autistic group had higher self-reported autistic traits, measured by the Autism-Spectrum Quotient 10-item (AQ-10; Ref.^56^) (*t*(59.203) = 8.801, *p* < 0.001). The autistic group had significantly lower baseline mood than the NA group (*t*(64) = 2.756, *p* = 0.008 < 0.01). Full details of the two groups are given in Table 3.

**Table 3:** Demographic details of the participants in online questionnnaire task.

	Autism	NA
*N* (male: female)	35 (11:24)	31 (7:24)
Age (years)	27.269 (6.106)	25.929 (4.562)
Verbal ability	76.714 (12.075)	74.548 (9.287)
Non-verbal ability	62.257 (21.056)	62.484 (18.156)
AQ-10^a^	7.286 (2.346)	3.032 (1.538)
PAS-10	2.809 (0.791)	3.374 (0.876)

Values are given as mean (standard deviation).

*NA* Non-autistic, *AQ* autism-spectrum quotient, *PAS-10* 10-item Positive affect schedule.

^a^Two autistic participants did not complete the AQ-10.

#### Laughter questionnaire

The 30-item Laughter Perception and Production Questionnaire (LPPQ) was a self-report questionnaire designed to explore people’s experiences of laughter in daily life. In terms of laughter production, ‘Frequency’ (Seven items) measures how often people produce laughter in daily life, and ‘Usage’ (Five items) measures people’s positive usage of laughter, particularly in using laughter as a social signal to mediate social context. In terms of laughter perception, ‘Understanding’ (Nine items) measures people’s understanding of the social meaning of other’s laughter; and ‘Liking’ (8 items) measures people’s general feelings towards laughter and their emotional valence of processing other’s laughter^37^.

#### Testing procedure

The LPPQ was presented within a longer testing battery for both in-lab and online participants, presented as printed questionnaires for in-lab testing, whilst the items were presented in random order on Gorilla Experiment Builder^59^ for online testing. In both settings, participants were asked to state the extent of their agreement with each item on a Likert-type scale with the following options: (1) (’strongly disagree’), (2) (‘moderately disagree’), (3) (‘slightly disagree’), (4) (‘neutral’), (5) (‘slightly agree’), (6) (‘moderately agree’), and (7) (‘strongly agree’).

### Ethics approval and consent to participate

Ethical approval for all projects was granted by the University College London Research Ethics Committee. All procedures performed in studies involving human participants were in accordance with the ethical standards of the institutional committee and with the 1964 Helsinki Declaration and its later amendments or comparable ethical standards. Informed written consent was obtained from participants prior to participation, and they were debriefed following completion.

### Supplementary Information

Supplementary Information.

## Data Availability

The anonymised data from the present study are available via the link: https://osf.io/8ft4x/?view_only=c632756d510b47269fa3d12cbc5a1da9.
