# Supplementary material for: Autistic adults perceive and experience laughter differently to non-autistic adults
Source: Sci Rep. 2024 May 21;14:11590. doi: 10.1038/s41598-024-56903-8 (PMC11109116; doi:10.1038/s41598-024-56903-8)
Supplement: Supplementary file 1 — Supplementary Information. [file 41598_2024_56903_MOESM1_ESM.pdf]

Supplementary Information

Autistic adults perceive and experience laughter differently to non-autistic adults

Ceci Q. Cai[1], Sarah J. White[1], Sinead H.Y. Chen[1], Marie A. E. Mueller[2], and Sophie K. Scott[1, *]

[1] Institute of Cognitive Neuroscience, University College London, London, WC1N 3AZ, United Kingdom

[2] Epidemiology & Applied Clinical Research, Division of Psychiatry, University College London, London, W1T 7BN, United Kingdom

[*] Email address: sophie.scott@ucl.ac.uk

Explicit ratings of laughter

Normality and Outliers. Considering our data is non-normally distributed, and we have a relatively small sample (see Figure S1), we used the median absolute deviation from the median (MAD) to detect outliers, which is more robust to detect outliers for non-normally distributed data and is immune to the sample size (Leys, Ley, Klein, Bernard, & Licata, 2013). We used 3.5 MAD as a threshold suggested by previous analysis rather than the more common three MAD, as we intended to include as much data as possible (Leys et al., 2013). Using this method, one NA participant was detected as an outlier on two rating scales (Valence/Arousal), one NA participant was detected as an outlier on three rating scales (Authenticity/Contagion/Valence), and one autistic participant was detected as an outlier on one rating scale (Valence). Full details of outliers are given in Table S1. As a result, three outliers were removed for the entire analysis of the explicit rating task. Secondly, some participants failed to give ratings due to the 3-second time limit for each response. Therefore, participants who missed more than 16 out of 40 rating trials (40%) on any rating scale were considered not attending to the task fully and were excluded from further data analysis on this rating scale. On this basis, one autistic participant, who missed 19 stimuli on the valence rating scale, was excluded from the data analysis on the valence rating scale.

Table S1. Details of the number of outliers removed and the distance between outliers and the selected threshold

		Authenticity		Contagion		Valence		Arousal	
		Genuine	Posed	Genuine	Posed	Genuine	Posed	Genuine	Posed
**NA**	High	8.085	5.735	8.150	5.335	7.507	6.285	7.876	5.476
	N	-	-	-	-	-	-	-	-
	Low	3.415	1.065	2.250	0.665	4.39	1.615	3.724	1.324
	N	1	-	1	-	2	-	1	-
	Distance	0.965		0.95		2.44/1.04		1.024	
**Autism**	High	8.085	5.826	8.179	6.907	8.195	6.495	7.466	6.629
	N	-	-	-	-	-	-	-	-
	Low	3.415	1.674	2.471	-0.357	3.005	1.305	3.834	0.921
	N	-	-	-	-	1	-	-	-
	Distance					0.105			

Note. Genuine = Ratings for genuine laughter; Posed = Ratings for posed laughter; N = Number of outlier(s); High = Median + 3.5xMAD; Low = Median - 3.5xMAD; Distance = distance between outliers and the selected threshold.

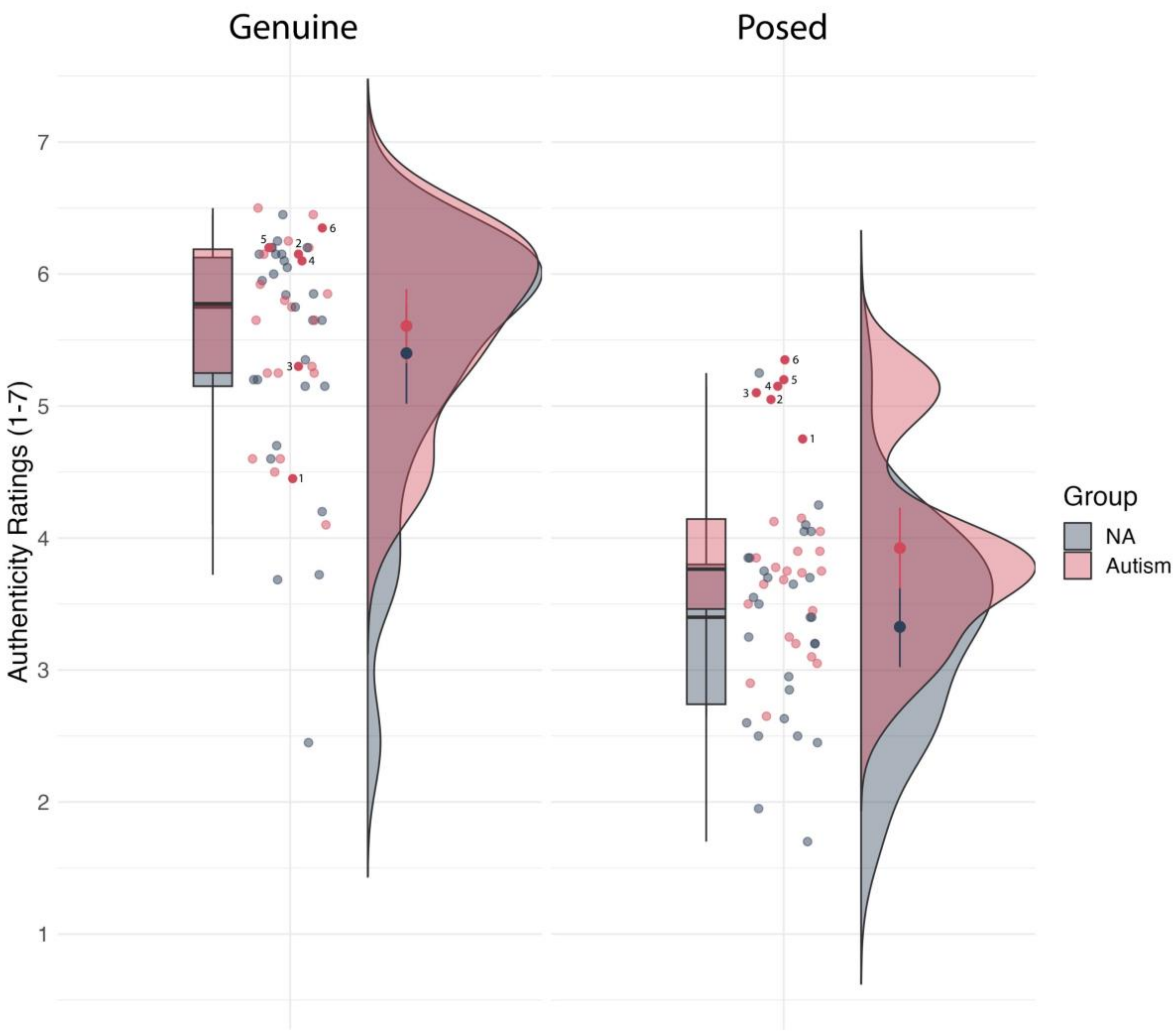
Genuine
Posed
Authenticity Ratings (1-7)
1
2
3
4
5
6
7
Group
NA
Autism

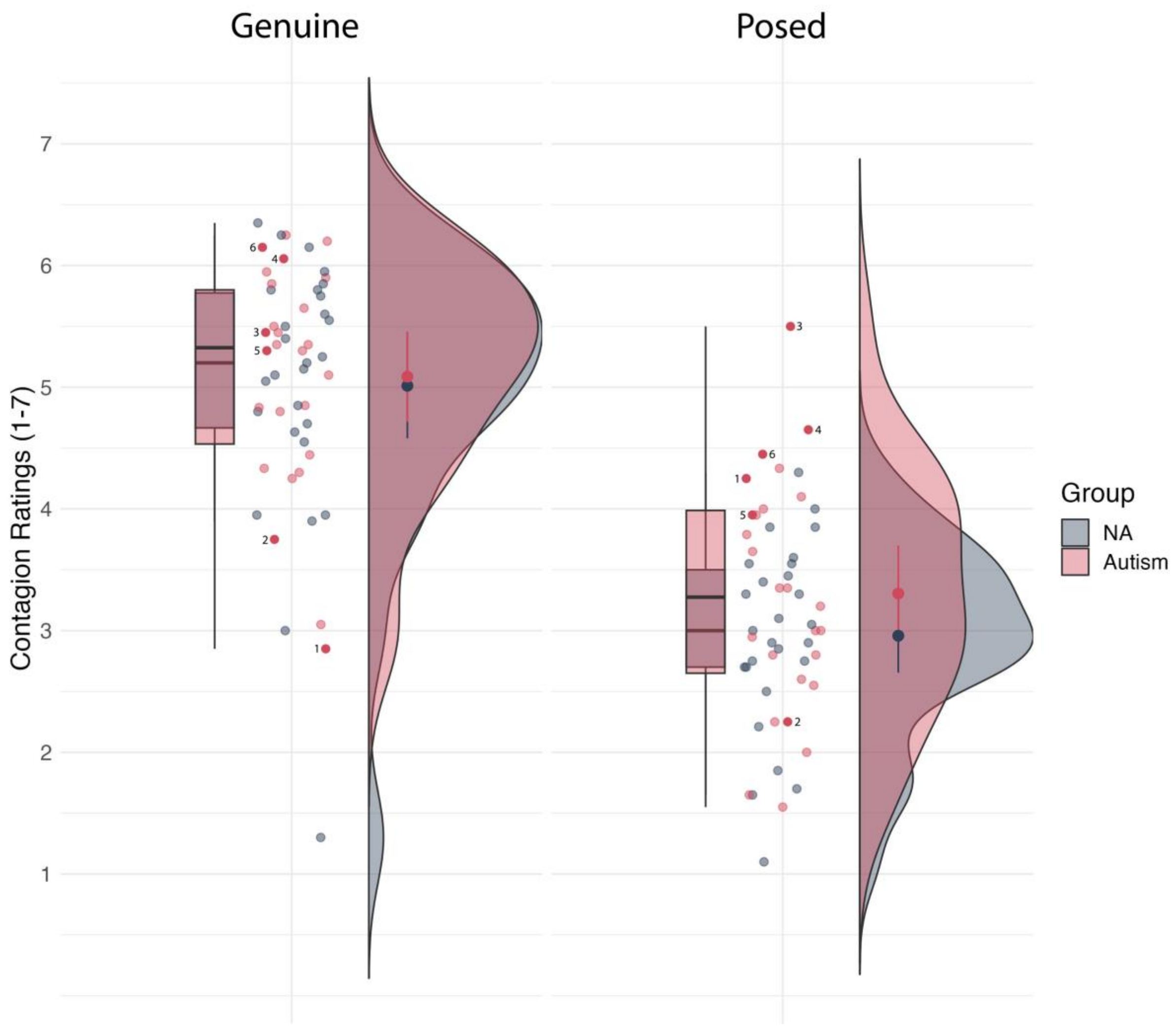
Genuine
Posed
Contagion Ratings (1-7)
7
6
5
4
3
2
1
Group
NA
Autism

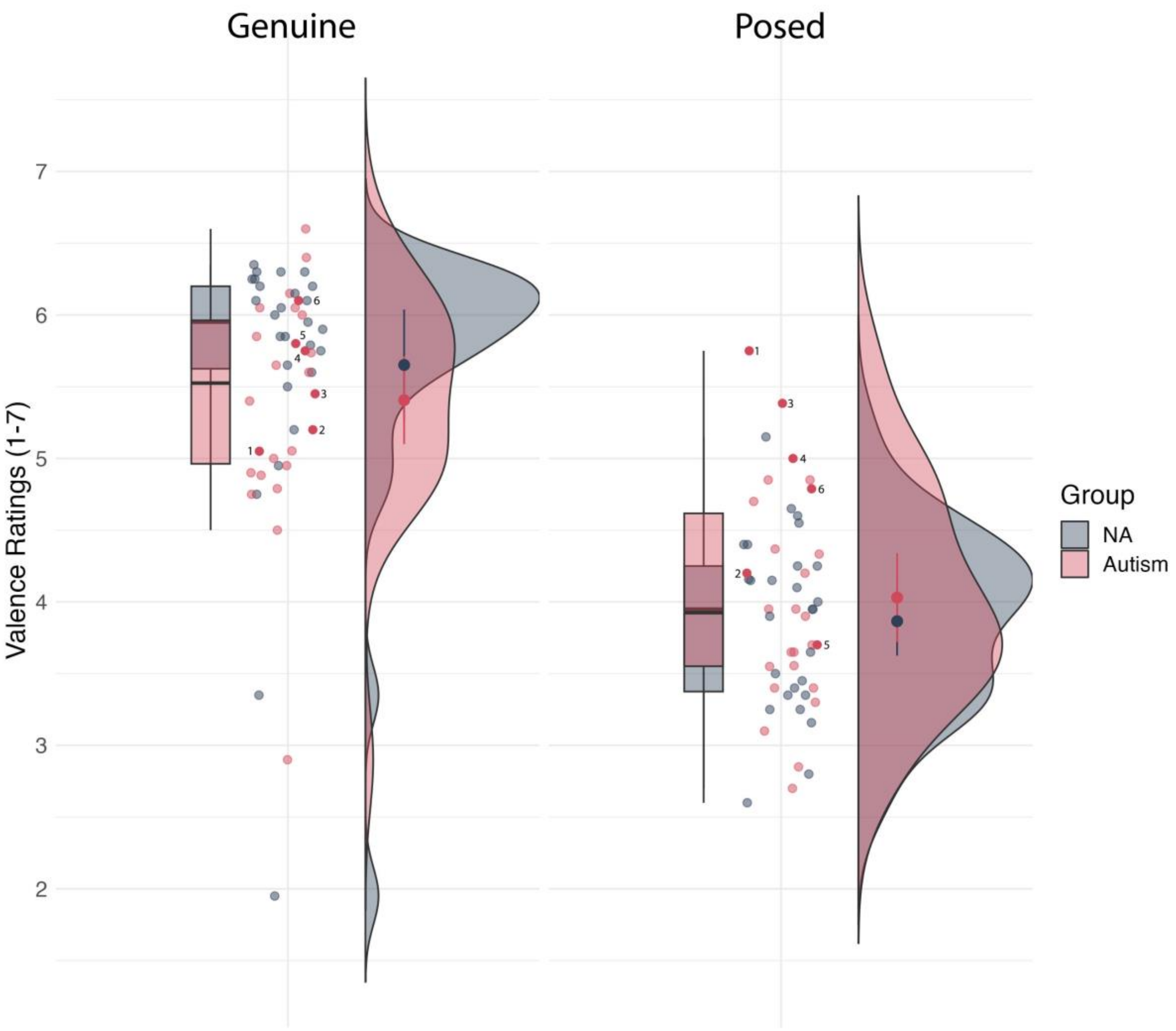
Genuine
Posed
Valence Ratings (1-7)
7
6
5
4
3
2
Group
NA
Autism

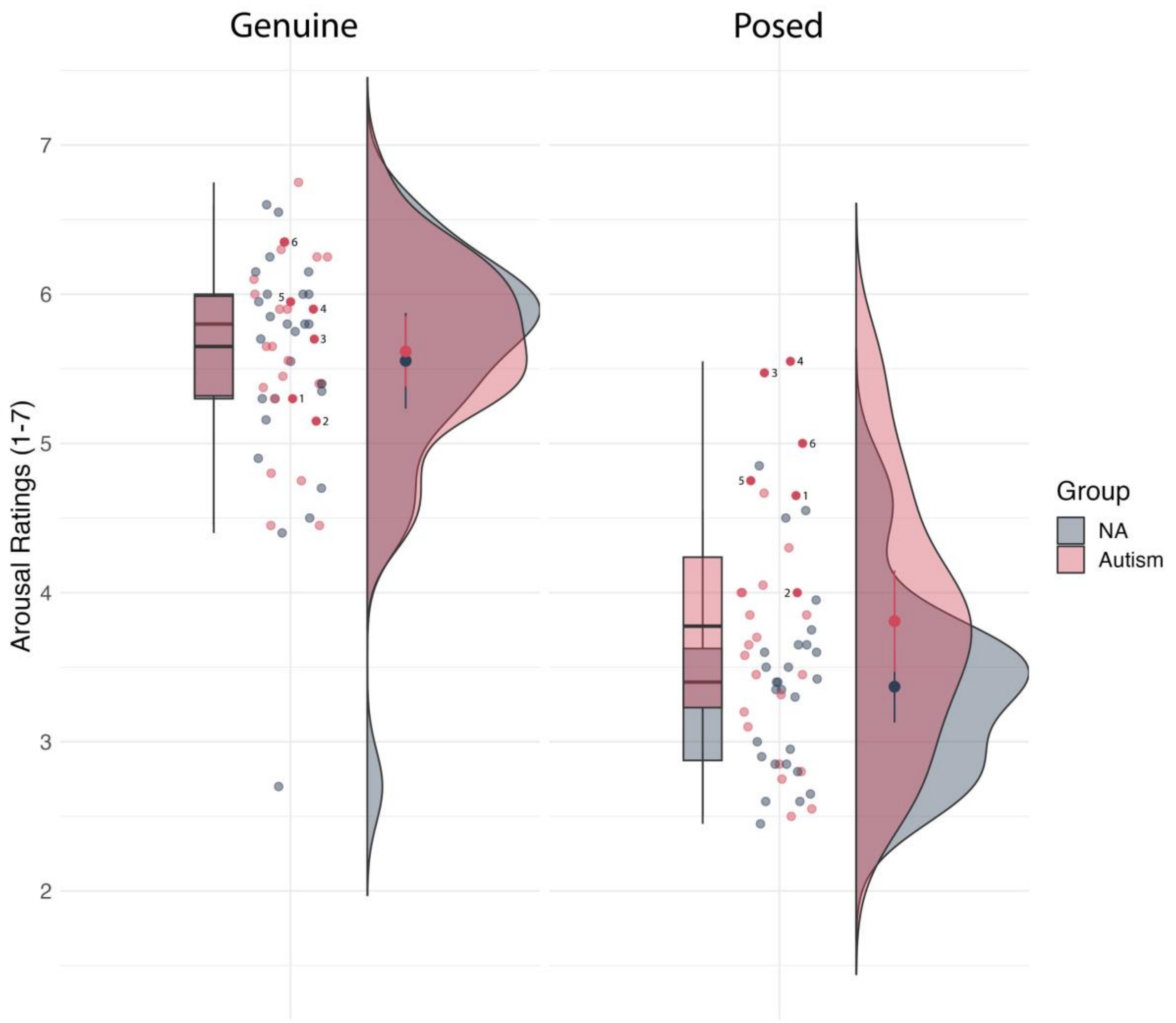

Figure S1. Rain-cloud plots and box plots of the distribution of ratings across all scales. Each light dot represents each participant's average rating of one type of laughter on each scale. Each dark dot with line = 1 ± SE. Since six participants gave usually high authenticity ratings on posed laughter, they have been identified on all graphs with a number next to their dot.

Principal Component Analysis on Explicit Ratings. The top principal component across the four ratings for both genuine and posed laughter was extracted using principal component analysis. The regressor for the ratings from genuine laughter

showed no significant difference between the autistic (*M* = .066, *SD* = .805) and non-autistic groups (*M* = .229, *SD* = .707), $t(47) = -.753$, $p = .455$, $d = .215$. In contrast, the regressor from posed laughter indicated that the ratings are close to being statistically significant between autistic (*M* = .256, *SD* = 1.091) and non-autistic groups (*M* = -.247, *SD* = .853), $t(47) = 1.802$, $p = .078$ ($p < .05$ one-tailed), $d = .515$.

Multiple regressions. Since we had only 40 observations, we selected the five acoustic features (mean pitch, median pitch, spectral centre of gravity, jitter (local), and harmonics-to-noise ratio (HNR)) that differed between genuine and posed laughter as predictors for the multiple linear regression. The regression model was applied to the average authenticity ratings of laughter for all participants, NA participants, and autistic participants. All regression models were significant. The predictors accounted for 57.9% of the variance in authenticity ratings from both NA and autistic participants, 58.2% for NA participants, and 53.4% for autistic participants. Among all acoustic features, only the spectral centre of gravity consistently predicted the authenticity ratings of laughter for all participants, NA participants, and autistic participants (see Table S2 for the full results).

Table S2. Beta-weights and adjusted R^2 values of the regression analyses of acoustic measures and authenticity ratings

Acoustic measure	All participants	NA	Autism
Pitch mean (Hz)	-.029	-.254	.267
Pitch median (Hz)	.452	.610	.228
Spectral centre of gravity (Hz)	**.389***	**.409***	**.348***
Jitter (local)	-.128	-.193	-.039
HNR (dB)	.046	.019	.075
Adjusted R^2	**.579******	**.582******	**.534******

Note. Significant predictors are highlighted in bold; * $p < .05$; *** $p < .001$.

Excluding Two Autistic Adults Below the ADOS Threshold and with AQ Scores Under 32. The explicit ratings were further analysed after excluding two autistic participants who were both below the ADOS threshold and had AQ scores below the cut-off point of 32: one scored 24 and the other did not complete the AQ.

On the authenticity rating, there was a significant main effect on the type of laughter, *F*[1,46] = 242.630, $p < .001$, η_p^2 = .841, indicating that participants found genuine laughter (*M* = 5.559, *SD* = .690) to be more authentic than posed laughter (*M* = 3.592, *SD* = .837). There was no significant main effect of group, *F*[1,46] = 2.739, *p* = .056, η_p^2 = .056, but there was a significant interaction effect between laughter type and group, *F*[1,46] = 7.542, $p < .01$, η_p^2 = .141. Post hoc analysis indicated that the non-autistic group (*M* = 3.289, *SD* = .791) rated posed laughter as significantly less authentic than the autism group (*M* = 3.921, *SD* = .773), *t*(46) = 2.793, $p < .01$. However, there was no significant difference for ratings of genuine laughter between non-autistic (*M* = 5.587, *SD* = .699) and autistic (*M* = 5.529, *SD* = .633) groups, *t*(46) = .285, *p* = .777.

On contagion rating, there was a significant main effect of type of laughter, *F*[1,46] = 217.611, $p < .001$, η_p^2 = .826, indicating that participants found genuine laughter (*M* = 5.084, *SD* = .843) to be more contagious than posed laughter (*M* = 3.080, *SD* = .900). There was no significant main effect of group, *F*[1,46] = .005, *p* = .944, η_p^2 = .000, and no significant interaction effect between laughter type and group, *F*[1,46] = 3.797, *p* = .057, η_p^2 = .076.

On valence rating, there was a significant main effect of type of laughter, *F*[1,45] = 202.670, $p < .001$, η_p^2 = .818, indicating that participants felt the sound of genuine laughter (*M* = 5.692, *SD* = .515) reflected a more positive emotion than the sound of posed laughter (*M* = 3.922, *SD* = .697). There was no significant main effect of group, *F*[1,45] = 1.922, *p* = .172, η_p^2 = .041, but there was a significant interaction effect

between laughter type and group, *F*[1,45] = 4.273, $p < .05$, $\eta_p^2 = .087$. Post hoc analysis indicated that the non-autistic group (*M* = 5.892, *SD* = .426) rated genuine laughter as significantly more positive than the autism group (*M* = 5.464, *SD* = .522), *t*(45) = 3.090, $p < .01$. However, there was no significant difference for ratings of posed laughter between non-autistic (*M* = 3.884, *SD* = .612) and autistic (*M* = 3.966, *SD* = .795) groups, *t*(45) = .397, *p* = .693.

On the arousal rating, there was a significant main effect on the type of laughter, *F*[1,46] = 335.982, $p < .001$, $\eta_p^2 = .880$, indicating that participants felt the sound of genuine laughter (*M* = 5.624, *SD* = .587) reflected stronger emotional arousal than the sound of posed laughter (*M* = 3.556, *SD* = .753). There was no significant main effect of group, *F*[1,46] = .600, *p* = .443, $\eta_p^2 = .013$, but there was a significant interaction between laughter type and group, *F*[1,46] = 4.263, $p < .05$, $\eta_p^2 = .085$. Post hoc analysis found there was no significant difference in ratings of posed laughter between non-autism (*M* = 3.387, *SD* = .612) and autism groups (*M* = 3.740, *SD* = .857), *t*(46) = 1.655, *p* = .105. Also, there was no significant difference in ratings of genuine laughter between non-autistic (*M* = 5.676, *SD* = .596) and autistic (*M* = 5.567, *SD* = .585) groups, *t*(46) = .643, *p* = .523.

Self-reported laughter questionnaire - LPPQ

Normality and Missing Data

In-lab dataset. Firstly, the missing data were examined in raw questionnaire data. In total, eight responses (0.2%) were missing from the complete dataset; further, the Little's Missing Completely at Random test (MCAR) was used, $\chi^2(113) = 138.254$, *p* = .053, and it indicated that the data were missing completely at random. Therefore, pairwise deletion was used to treat the missing data; each missing data point was excluded from the mean calculations of the composite scores for each component.

Secondly, negatively phrased items were reversed and the composite scores for each component were calculated by averaging the score of the contributing items, resulting in a Liking, Understanding, Usage and Frequency score for each participant, ranging from 1 to 7. For example, for the component Understanding, the items were coded in a way that a higher composite score corresponds with a better understanding of other people's laughter. In the same way, the other three components were coded: higher composite scores correspond to higher Frequency of laughter, more Liking of laughter, and more Use of laughter.

The distribution of each composite score among the NA and autism groups was assessed for its normality. The Shapiro-Wilk test indicated that the data of the NA group was normally distributed on Frequency, $W(67) = .972$, $p = .137$, Understanding, $W(67) = .982$, $p = .457$, and Usage, $W(67) = .975$, $p = .200$; and the data of the autism group was normally distributed on all four components: Frequency, $W(28) = .972$, $p = .647$, Understanding, $W(28) = .964$, $p = .421$, Usage, $W(28) = .972$, $p = .622$ and Liking, $W(28) = .940$, $p = .108$. Although one component, Liking, was not normally distributed in the NA group, $W(67) = .929$, $p < .001$, the Q-Q plot suggested that the data was approximately normally distributed. In addition, one potential outlier was detected on the Liking component in each group. As these two outliers did not alter statistical outcomes, all data were included in further analyses. See Figure S2 for details.

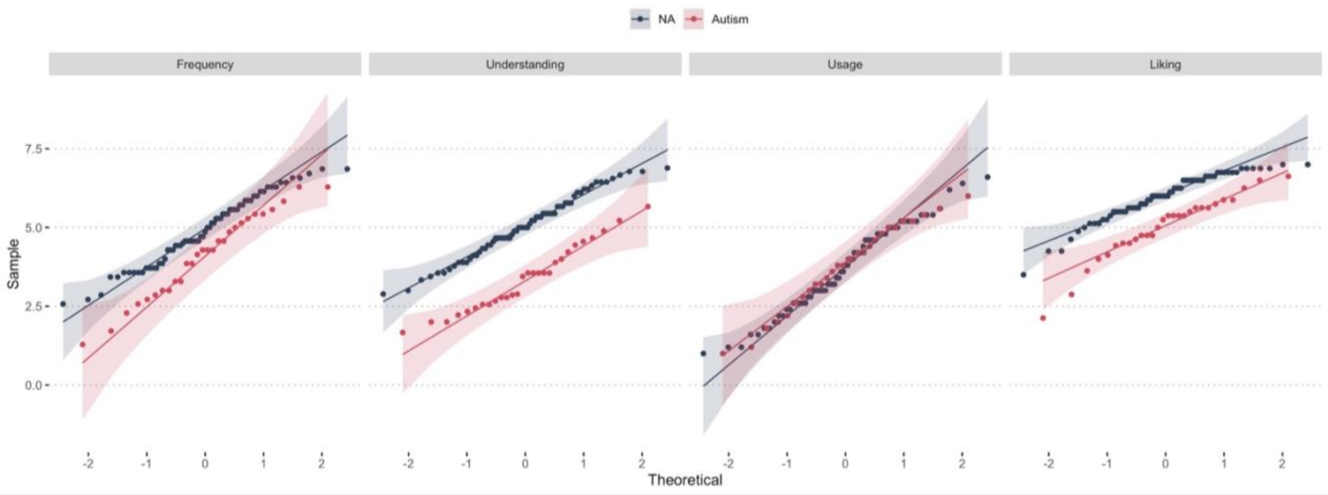

Figure S2. Q-Q plots for ratings on four components in the Non-autistic and Autistic groups from the in-lab dataset

Online dataset. The composite scores of the components were calculated for each participant based on the method above. The distribution of the composite score of each component among the NA and autism groups was assessed for its normality. The Shapiro-Wilk test indicated that the data of the NA group was normally distributed on Frequency, $W(52) = .979$, $p = .492$, and Understanding, $W(52) = .977$, $p = .420$; and the data of the autism group was normally distributed on Frequency, $W(35) = .943$, $p = .071$, Understanding, $W(35) = .973$, $p = .543$, and Liking, $W(35) = .974$, $p = .559$. Although the NT group was not normally distributed on two components, Liking, $W(52) = .947$, $p = .021$, and Usage, $W(52) = .953$, $p = .040$, and the autism group was not normally distributed on Usage, $W(35) = .924$, $p = .019$, the Q-Q plot suggested that the data was approximately normally distributed. In addition, two potential outliers were detected in the Usage component in the autism group. As these two outliers did not alter statistical outcomes in the results of the matched groups, therefore, all data were included in further analyses. See Figure S3 for details.

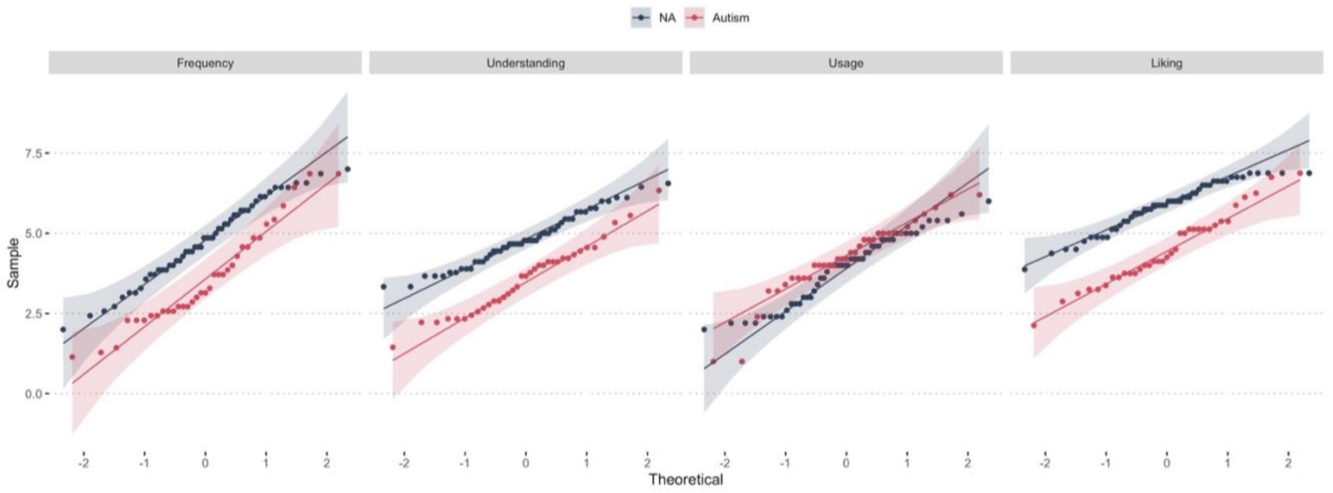

Figure S3. Q-Q plots for ratings on four components in the Non-autistic and Autistic groups from online dataset

Group differences in the loading items on four components.

We further explore how the items loaded in each component differ between the two groups from both in-lab and online dataset.

Table S3 In-lab group difference in loading items

	t	*p*
Frequency (F)		
I rarely laugh when I am on my own.	1.81	.077
I have a subdued laugh.	1.73	.090
I find things funny, but I rarely laugh out loud.	2.00	.051
I laugh less often than most people I know.	2.90	.005
I rarely break into uncontrollable laughter.	.69	.496
If I find something funny, I often laugh out loud.	3.49	.000
I laugh more than most people I know.	1.36	.176
Understanding (UN)		
I can never tell if someone is laughing because they want something from me.	4.43	.000
Sometimes I find it difficult to tell when someone is laughing just to be polite.	4.91	.000
I can always tell if someone is laughing at or with me.	4.62	.000

I can tell when people are laughing because they want something from me.	2.81	.007
I can tell when someone is laughing to stop me getting angry at them.	2.67	.010
Sometimes I find it difficult to tell when someone is laughing nastily.	4.63	.000
I can never tell if someone is deliberately laughing to pretend that they are amused.	4.20	.000
I can never tell if someone is laughing to stop me getting angry with them.	5.63	.000
I can tell when someone is deliberately laughing to pretend that they are amused.	3.09	.003
Usage (US)		
I laugh more when I want people to like me.	-.54	.589
I often laugh to avoid expressing frustration.	-.76	.453
I often laugh deliberately to show that I like someone.	2.91	.005
Sometimes I laugh to stop other people from getting angry with me.	-.85	.398
I sometimes laugh to avoid expressing sadness.	-.40	.690
Liking (L)		
Hearing laughter makes me nervous.	3.32	.002
I dislike people who laugh a lot.	2.75	.008
When I am upset, hearing someone laugh makes me feel better.	-.14	.886
I enjoy the sound of people laughing.	2.32	.024
Laughter has a positive influence on interactions with people.	3.33	.002
I find laughter an important part of intimate relationships.	3.18	.003
If I am happy, hearing someone laugh makes me even happier.	2.67	.012
A friend's laughter is always good to hear.	3.52	.000
N/A item		
Hearing people faking laughter irritates me.	1.78	.080

Note. The scoring of these underlined items is reversed. Independent t-tests were conducted on each item's average ratings for non-autistic versus autistic individuals.

Table S4 Online group difference in loading items

	t	*p*
Frequency (F)		
I rarely laugh when I am on my own.	1.97	.054
I have a subdued laugh.	2.28	.026
I find things funny, but I rarely laugh out loud.	2.71	.009
I laugh less often than most people I know.	4.55	.000

I rarely break into uncontrollable laughter.	2.59	.012
If I find something funny, I often laugh out loud.	3.10	.003
I laugh more than most people I know.	3.43	.001
Understanding (UN)		
I can never tell if someone is laughing because they want something from me.	4.63	.000
Sometimes I find it difficult to tell when someone is laughing just to be polite.	2.58	.012
I can always tell if someone is laughing at or with me.	5.72	.000
I can tell when people are laughing because they want something from me.	1.79	.078
I can tell when someone is laughing to stop me getting angry at them.	2.27	.027
Sometimes I find it difficult to tell when someone is laughing nastily.	4.29	.000
I can never tell if someone is deliberately laughing to pretend that they are amused.	3.49	.000
I can never tell if someone is laughing to stop me getting angry with them.	3.13	.003
I can tell when someone is deliberately laughing to pretend that they are amused.	3.38	.001
Usage (US)		
I laugh more when I want people to like me.	-1.63	.110
I often laugh to avoid expressing frustration.	-2.68	.009
I often laugh deliberately to show that I like someone.	.28	.782
Sometimes I laugh to stop other people from getting angry with me.	-.52	.604
I sometimes laugh to avoid expressing sadness.	1.34	.185
Liking (L)		
Hearing laughter makes me nervous.	5.18	.000
I dislike people who laugh a lot.	3.90	.000
When I am upset, hearing someone laugh makes me feel better.	3.09	.003
I enjoy the sound of people laughing.	5.41	.000
Laughter has a positive influence on interactions with people.	7.45	.000
I find laughter an important part of intimate relationships.	4.72	.000
If I am happy, hearing someone laugh makes me even happier.	3.92	.000
A friend's laughter is always good to hear.	4.63	.000
N/A item		
Hearing people faking laughter irritates me.	-3.40	.001

Note. The scoring of these underlined items is reversed. Independent t-tests were conducted on each item's average ratings for non-autistic versus autistic individuals.

Explicit ratings of laughter and self-reported LPPQ

Bimodal Subgroups Performance in Explicit Ratings and In-lab LPPQ

Two autistic subgroups were detected from the authenticity ratings. The distribution of subgroup one (N=6) is detailed across four ratings (Figure S1). Further analysis revealed that subgroup one showed no significant differences in authenticity ($t(5) = 1.799$, $p = .132$), contagion ($t(5) = 1.500$, $p = .194$), and valence ($t(5) = 1.908$, $p = .115$) ratings. However, a significant difference was observed in arousal ratings ($t(5) = 4.218$, $p < .01$). Conversely, subgroup two (N=19) exhibited significant differences in authenticity ($t(18) = 11.078$, $p < .001$), contagion ($t(18) = 11.523$, $p < .001$), valence ($t(17) = 9.013$, $p < .001$), arousal ($t(18) = 11.402$, $p < .001$) ratings.

For the self-reported LPPQ, only one autistic participant from subgroup one and 11 autistic participants from subgroup two completed the in-lab testing. After excluding subgroup one, a significant difference was observed between the autism and NA groups on Frequency, $t(55) = 2.573$, $p < .05$, Understanding, $t(55) = 6.093$, $p < .001$, and Liking, $t(42.4) = 3.942$, $p < .001$, but not on Usage, $t(55) = .022$, $p = .983$. Similar results were found after excluding subgroup two, there was a significant difference between the autism and NA group on Frequency, $t(45) = 2.353$, $p < .05$, Understanding, $t(45) = 4.721$, $p < .001$, and Liking, $t(21.55) = 3.373$, $p < .01$, but not on Usage, $t(45) = -.838$, $p = .406$.

Overlapped in-lab small subsample for correlation analysis

Table S5 Background details of the overlapped subsample

	NA	Autism	P value
N (male: female)	11 (7:4)	11 (9:2)	.338
Age (years)	32.636 (6.712)	34.727 (4.519)	.402
Verbal IQ	121.273(8.380)	116.364 (14.459)	.344
Full Scale IQ	120.273 (11.136)	117.000 (14.880)	.566
AQ ***	14.909 (6.625)	31.909 (10.281)	<.001
BDI	6.636 (7.075)	12.727 (7.390)	.062

Note: Values are given as mean (standard deviation). NA = Non-autistic.
